# A review on the role of miRNA-324 in various diseases

**DOI:** 10.3389/fgene.2022.950162

**Published:** 2022-08-10

**Authors:** Sepideh Kadkhoda, Bashdar Mahmud Hussen, Solat Eslami, Soudeh Ghafouri-Fard

**Affiliations:** ^1^ Department of Medical Genetics, School of Medicine, Tehran University of Medical Sciences, Tehran, Iran; ^2^ Department of Pharmacognosy, College of Pharmacy, Hawler Medical University, Erbil, Iraq; ^3^ Center of Research and Strategic Studies, Lebanese French University, Erbil, Iraq; ^4^ Department of Medical Biotechnology, School of Medicine, Alborz University of Medical Sciences, Karaj, Iran; ^5^ Dietary Supplements and Probiotic Research Center, Alborz University of Medical Sciences, Karaj, Iran; ^6^ Department of Medical Genetics, Shahid Beheshti University of Medical Sciences, Tehran, Iran

**Keywords:** miR-324, cancer, biomarker, expreesion, prognostic

## Abstract

Recent studies have revealed important functions of several microRNAs (miRNAs) in the pathogenesis of human diseases. miR-324 is an example of miRNAs with crucial impacts on the pathogenesis of a wide range of disorders. Gene ontology studies have indicated possible role of miR-324 in responses of cells to the leukemia inhibitory factor, long-term synaptic potentiation, positive regulation of cytokines production and sensory perception of sound. In human, miR-324 is encoded by *MIR324* gene which resides on chromosome 17p13.1. In the current manuscript, we provide a concise review of the role of miR-324 in the pathogenesis of cancers as well as non-cancerous conditions such as aneurysmal subarachnoid hemorrhage, diabetic nephropathy, epilepsy, pulmonary/renal fibrosis, ischemic stroke and ischemia reperfusion injuries. Moreover, we summarize the role of this miRNA as a prognostic marker for malignant disorders.

## Introduction

MicroRNAs (miRNAs) are small regulatory molecules that have principal roles in several cellular processes. They DNA sequences coding these transcripts are mainly transcribed into primary miRNAs. Primary miRNAs are then processed into the precursor and mature miRNAs through a multistep process. miRNAs principally affect genes expression through binding with the 3′ UTR of the target mRNAs. This interaction induces degradation of mRNAs or repression of their translation. In some cases, miRNAs can interact with 5′ UTR or even coding or promoter regions (O'Brien et al., 2018). Notably, the interplay between miRNAs and their targets is dynamic. It depends on subcellular localization of miRNAs, quantities of both miRNAs and target transcripts, and the affinity of miRNA-target interaction which is mainly determined by the mode of base pairing (O'Brien et al., 2018).

miR-324 is an example of miRNAs with crucial impacts on the pathogenesis of human disorders. This miRNA participates in gene silencing. Gene ontology studies have indicated possible role of miR-324 in diverse processes, namely responses of cells to the leukemia inhibitory factor, long-term synaptic potentiation, positive regulation of cytokines production and sensory perception of sound (https://www.ncbi.nlm.nih.gov/gene/442898). In human, miR-324 is encoded by *MIR324* gene which is located on chromosome 17p13.1.

Through interaction with a variety of RNA molecules, miR-324 participates in the etiopathology of several cancers as well as non-cancerous disorders such as aneurysmal subarachnoid hemorrhage, anorectal malformation, cardiac diseases, diabetic nephropathy, epilepsy, HIV lipodystrophy, idiopathic pulmonary fibrosis, ischemic stroke, myocardial ischemia reperfusion (I/R) injuries, nerve injury, osteoarthritis, Parkinson’s disease, polycystic ovarian syndrome, renal fibrosis and thermal injury.

In the current manuscript, we provide a concise review of the role of miR-324 in the pathogenesis of mentioned disorders. Moreover, we summarize the role of this miRNA as a prognostic marker for malignant disorders.

## Role of miR-324 in cancers

### Cervical cancer

Cervical cancer is an example of cancers in which miR-324 is dysregulated. However, different studies in this type of cancer have reported inconsistent results regarding the pattern of expression and exact role of miR-324 in cervical cancer. This inconsistency might be explained by different roles of miR-324-5p and miR-324-3p. Zhang et al. (2020) have shown that upregulation of the cytoplasmic long non-coding RNA (lncRNA) LINC00511 leads to downregulation of miR-324 in cervical cancer cell lines. DRAM1 has been identified as a target of miR-324 in these cells. Both miR-324-5p mimics and LINC00511 targeting siRNAs reverse the oncogenic effects of DRAM1 on cervical cancer cells. Cumulatively, LINC00511 has been found to act as a competing endogenous RNA (ceRNA). This lncRNA regulates activity of miR-324-5p/DRAM1 axis and promotes progression of cervical cancer irrespective of the presence of HPV (Zhang et al., 2020). Similarly, through gain- and loss- experiments, Jiang et al. have shown that miR-324-5p inhibits colony construction, proliferative ability, migration, invasive properties and epithelial-mesenchymal transition (EMT) in cervical cancer. The sponging effect of TPT1-AS1 on miR-324-5p leads to enhancement of cell growth and metastases in this type of cancer (Jiang et al., 2018).

**FIGURE 1:**
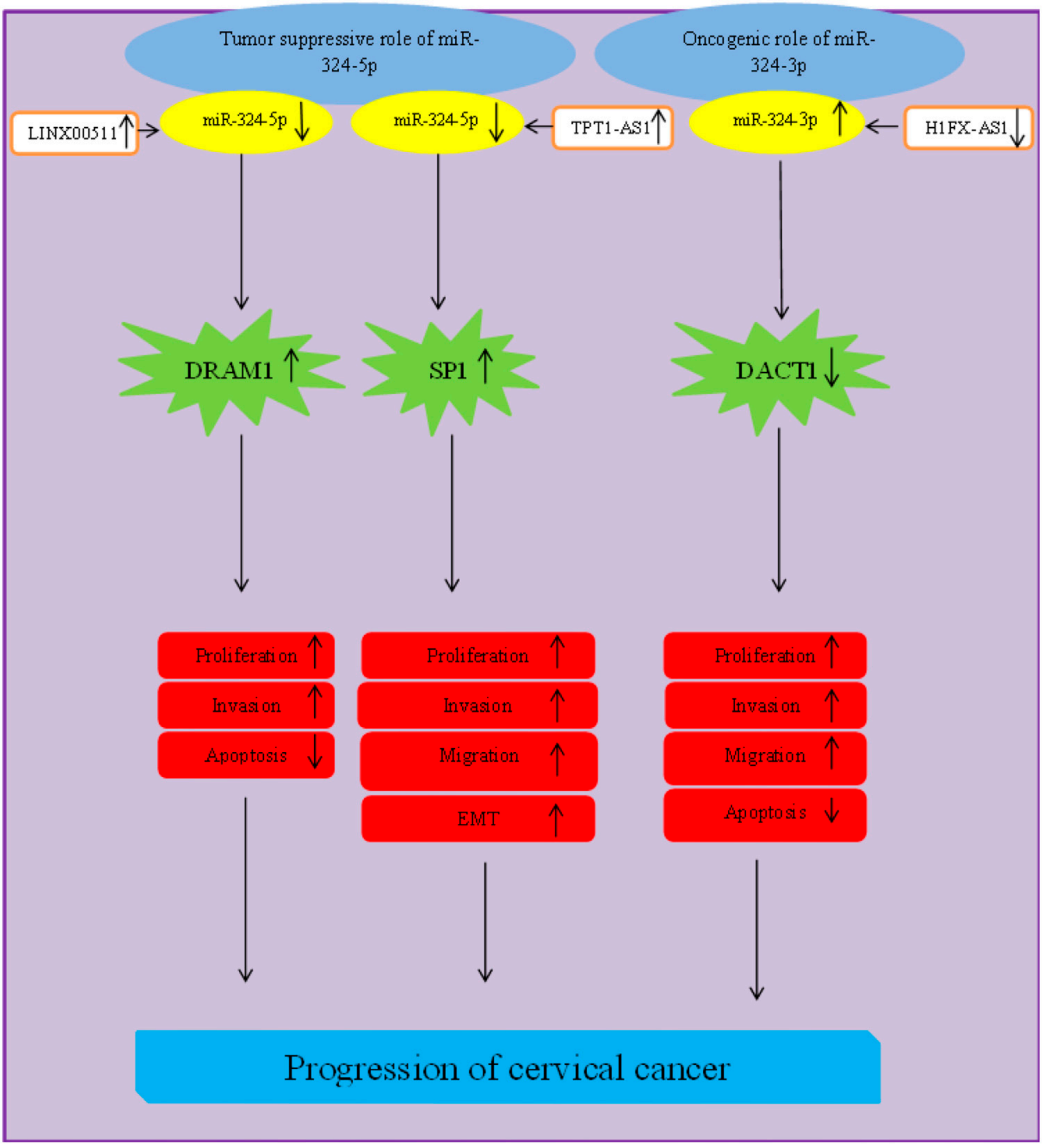
Role of miR-324 in progression of cervical cancer.

Conversely, Shi et al. (2020) have reported upregulation of miR-324 in cervical cancer cells and clinical samples. They have also shown that the tumor suppressor lncRNA H1FX-AS1 acts as a ceRNA for miR-324-3p to surge expression of DACT1 (Shi et al., 2020). Figure 1 shows the impact of miR-324 in cervical carcinogenesis.

### Colorectal cancer

**FIGURE 2:**
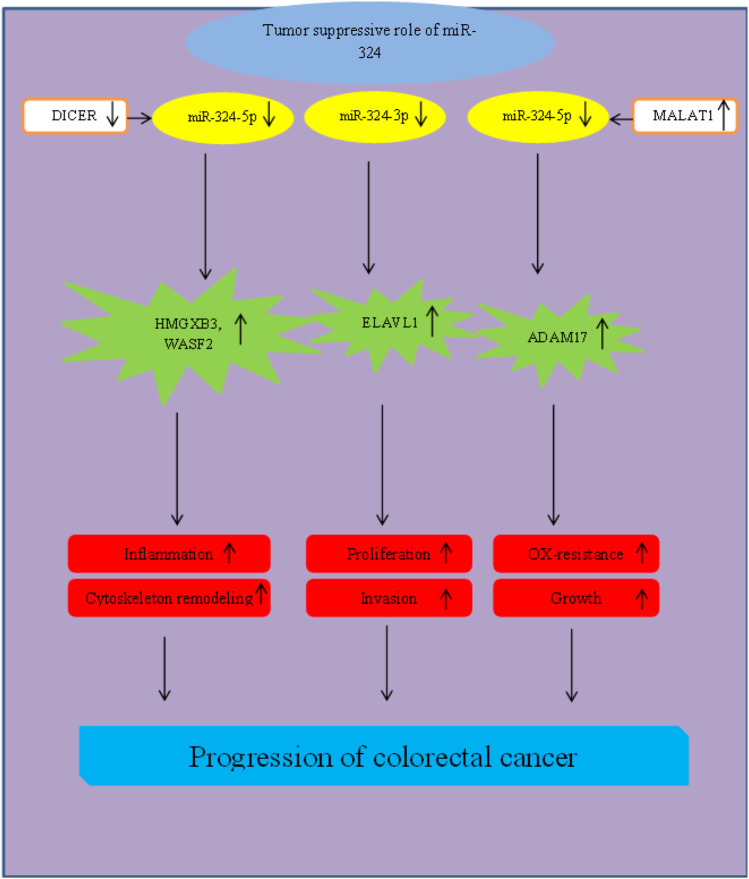
Role of miR-324 in progression of colorectal cancer.

In colorectal cancer, both miR-324-5p (Huang et al., 2022) and miR-324-3p (Xiao et al., 2019) have been found to be downregulated. An *in vitro* study in colorectal cancer cells has shown the sponging effects of VPS9D1-AS1 on miR-324-5p and demonstrated this mechanism as the underlying cause of downregulation of miR-324-5p in these cells. In line with the observed targeting of ITGA2 3′ UTR by miR-324-5p targets, miR-324-5p knock-down or forced over-expression of ITGA2 has reduced the impact of VPS9D1-AS1 silencing in colorectal cancer cells. Taken together, VPS9D1-AS1/miR-324-5p/ITGA2 axis has been reported to affect pathogenesis of colorectal cancer (Huang et al., 2022). Another study has confirmed the role of Dicer in the regulation of expression of miR-324. Assessment of expression profile of colorectal cancer cell lines as well as intestinal epithelial cells of mice has shown significant reduction of miR-324-5p expression after deletion of Dicer. miR-324-5p has been shown to bind to the 3′ UTRs of HMGXB3 and WASF-2, two important molecules with essential roles in cell motility and cytoskeleton remodeling. Intraperitoneal administration of a miR-324-5p agonist has reduced chronic inflammatory responses and cytoskeleton remodeling of colorectal epithelial cells and reestablished intestinal barrier integrity in Dicer-deleted cells of mice. Cumulatively, DICER/miR-324-5p/HMGXB3/WASF-2 axis has been found to affect colorectal tumorigenesis through modulation of cytoskeleton remodeling and intestinal barrier function (Sun et al., 2017). MALAT1/miR-324-3p/ADAM17 (Fan et al., 2020) and miR-324-3p/ELAVL1 (Xiao et al., 2019) axes are two other molecular axes participating in the pathogenesis of colorectal cancer (Figure 2).

### Gastric cancer

**FIGURE 3:**
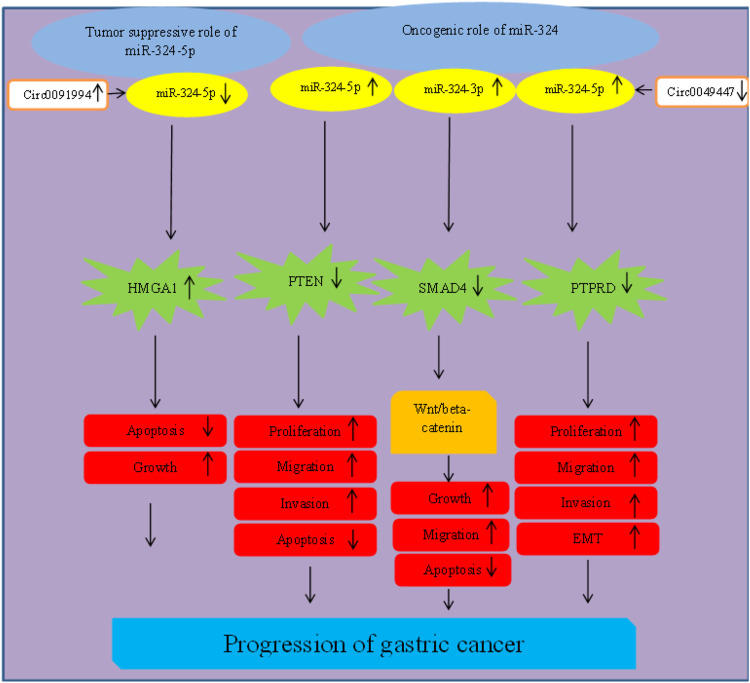
Role of miR-324 in progression of gastric cancer.

In gastric cancer, two different studies have reported upregulation of miR-324-5p (Tang et al., 2021a; Zheng et al., 2021), while another study has reported downregulation of this miRNA (Xie et al., 2021). miR-324-3p has also been reported to be upregulated in gastric cancer tissues (Sun et al., 2018). Circ0049447/miR-324-5p (Tang et al., 2021a) and Circ0091994/miR-324-5p/HMGA1 (Xie et al., 2021) axes have been shown to affect gastric tumorigenesis (Figure 3).

### Brain tumors

**FIGURE 4:**
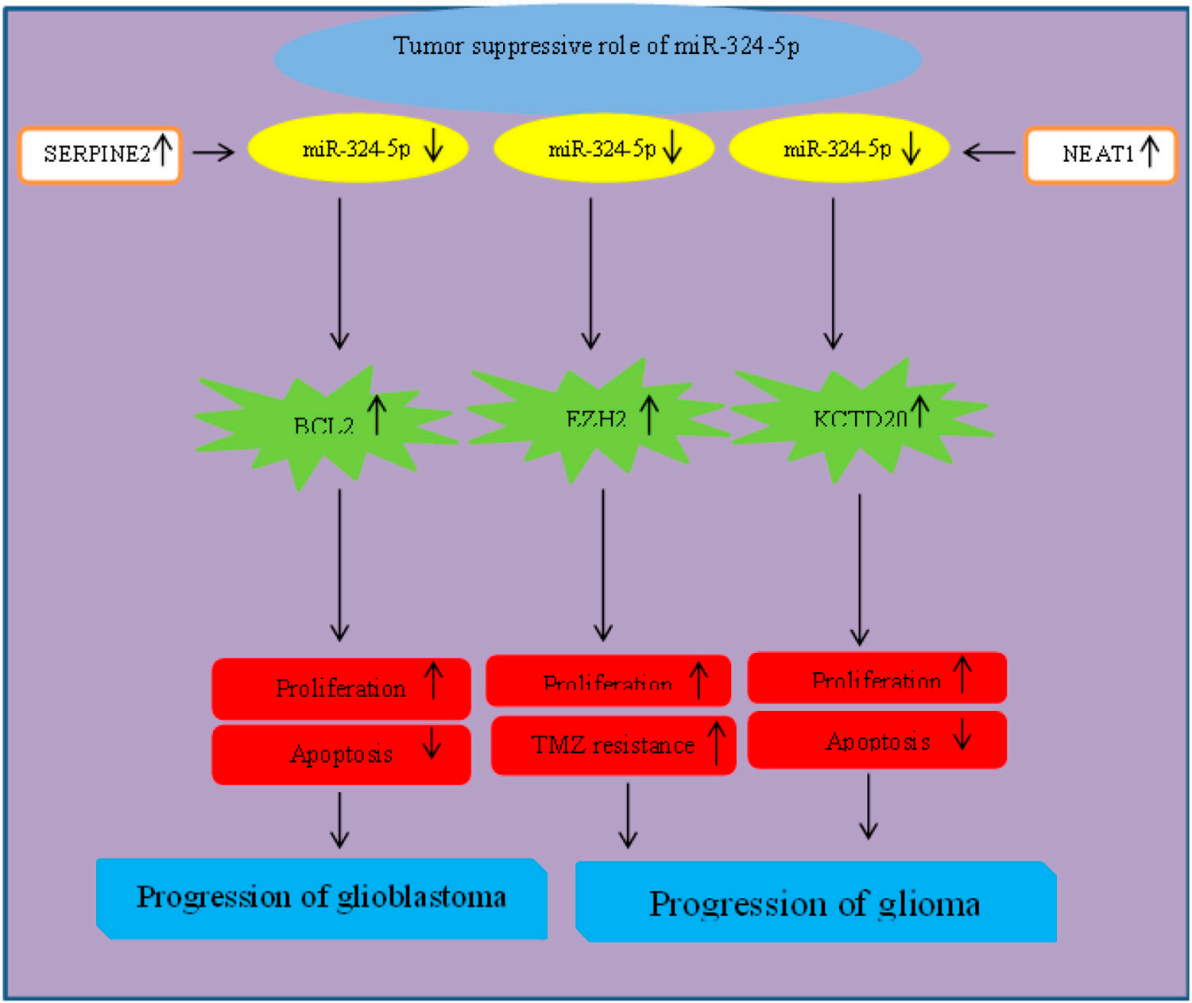
Role of miR-324 in progression of brain tumors.

Different experiments in brain tumors have confirmed tumor suppressor role of miR-324. For instance, SERPINE2 has been found to induce proliferation of glioblastoma cells and inhibit their apoptosis *via* influencing activity of miR-324-5p/BCL2 axis (Li et al., 2021). Moreover, the oncogenic lncRNA NEAT1 promotes progression of glioma through sponging miR-324-5p and inducing expression of KCTD20 (Zhang et al., 2021). Finally, miR-324-5p could hamper cell proliferation and Temozolomide (TMZ) resistance *via* targeting EZH2 (Zhi et al., 2017) (Figure 4).

### Hepatocellular carcinoma

**FIGURE 5:**
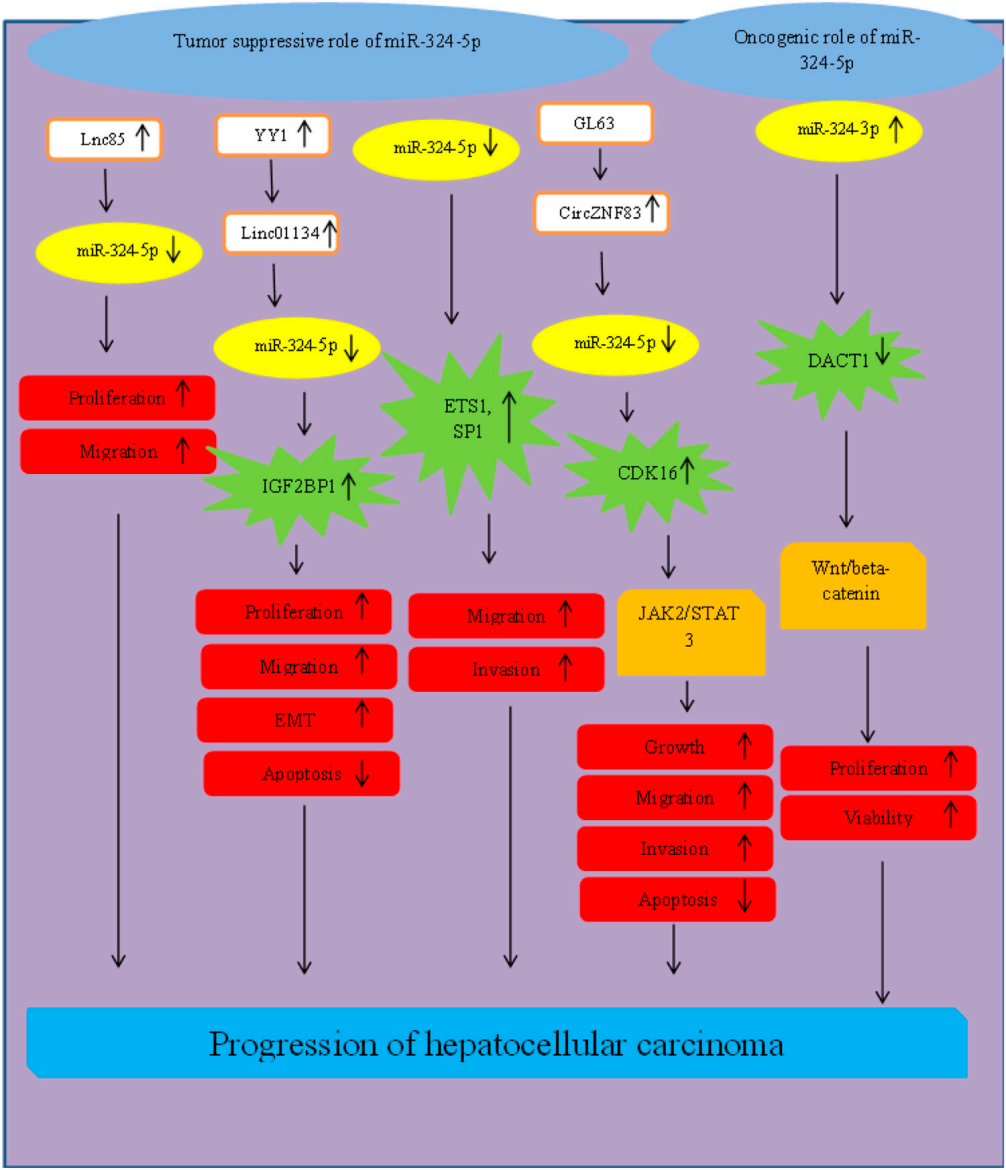
Role of miR-324 in progression of hepatocellular carcinoma.

In hepatocellular carcinoma, miR-324-5p has been shown to be downregulated (Huang et al., 2020a), while miR-324-3p has been upregulated (Tuo et al., 2017). Comprehensive assessment of expression profile of plasma exosomes in patients with hepatocellular carcinoma has led to identification of a novel differentially expressed lncRNA, namely RP11-85G21.1 (lnc85) which has been shown to promote proliferation and migration of hepatocellular carcinoma cells through binding with miR-324-5p (Huang et al., 2020a). CircZNF83/miR-324-5p/CDK16 (Zhao et al., 2021a), LINC00491/miR-324-5p/ROCK1 (Wan g et al., 2021) and YY1/linc01134/miR-324-5p/IGF2BP1 (Rong et al., 2020) are other molecular axes that influence pathogenesis of this type of cancer (Figure 5).

### Other types of cancer

**FIGURE 6:**
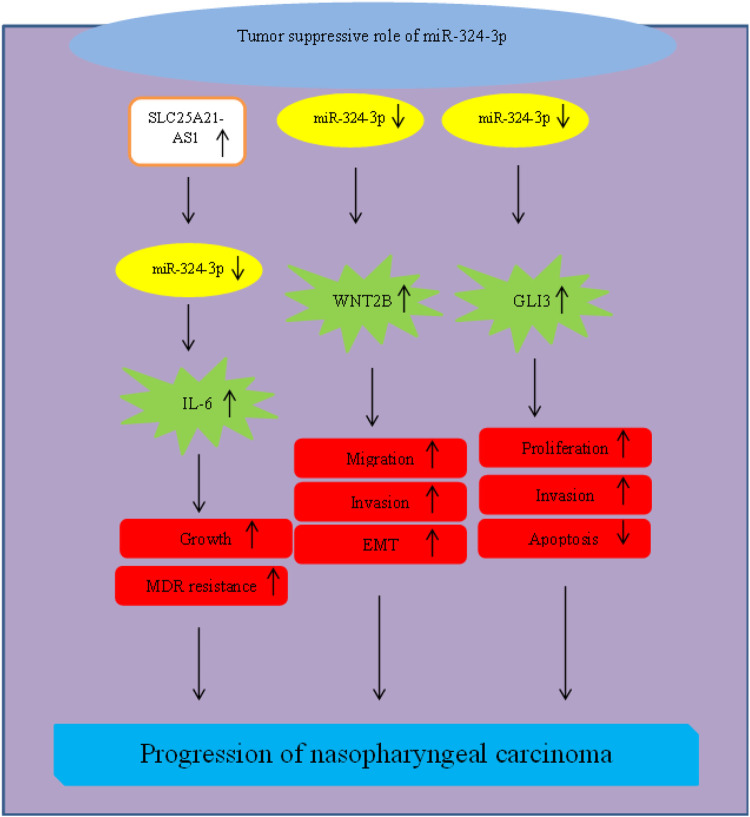
Role of miR-324 in progression of nasopharyngeal cancer.

In nasopharyngeal carcinoma, miR-324-3p has been shown to exert a tumor suppressor role. This miRNA could exert this effect through targeting GLI3 (Zhang et al., 2017), WNT2B (Liu et al., 2017) and SMAD7 (Xu et al., 2015). Moreover, the oncogenic role of SLC25A21-AS1 in this type of cancer has been found to be exerted through sponging miR-324-3p and increasing expression of IL-6 (Wang et al., 2020a) (Figure 6).

Studies in other types of cancers have also indicated importance of miR-324 in the evolution and progression of cancer (Table 1).

**TABLE 1:** miR-324 in cancers (ANCS, adjacent non-cancerous sample; OX, oxaliplatin; TMZ, temozolomide; MDR, multidrug resistance; FNA, fine needle aspiration; LNM, lymph node metastasis).

Tumor type	Expression	Samples	Downstream targets	Upstream molecules	Description	References
Bladder cancer (BC)	miR-324 (3p, 5p) upregulated in TCGA database, miR-324-5p upregulated in 22 BC tissues versus 13 ANCSs	TCGA database, 22 BC tissues and 13 ANCSs	PTPRD, RCAN1, AMHR2, EYA4, UNC5C, SETBP1,CDC42BPA, APOLD1 and GPX3	—	miR-324 (3p, 5p) upregulation could promote proliferation, colony formation ability, and invasiveness.	Tsai et al. (2020)
Breast cancer	miR-324-3p (down)	45 pairs of cancer tissues and ANCSs/mice	ACK1	LINC00963	LINC00963 *via* miR-324-3p/ACK1 axis could promote malignancy features and radioresistance.	Zhang et al. (2019a)
Triple-negative breast cancer (TNBC)	miR-324-3p (down)	70 pairs of TNBC tissues and ANCSs/mice	SUDS3, caspase3	SNHG22	SNHG22 *via* miR-324-3p/SUDS3 can promote tumorigenesis.	Fang et al. (2020)
Cervical cancer (CC)	miR-324-5p (down)	GEPIA database, 19 pairs of CC tissues and ANCSs	DRAM1	LINC00511	LINC00511 *via* miR-324-5p/DRAM1 axis could provoke tumorigenesis.	Zhang et al. (2020)
	miR-324-3p (up)	50 pairs of CC tissues and ANCSs/mice	DACT1, cleaved Caspase 3, and Bcl-2	H1FX-AS1	H1FX-AS1 through miR-324-3p/DACT1 axis could block cell proliferation.	Shi et al. (2020)
	miR-324-5p (down)	40 pairs of CC tissues and ANCSs/mice	SP1, E-cadherin, vimentin	TPT1-AS1	TPT1-AS1via miR-324-5p/SP1 could promote tumorigenesis.	Jiang et al. (2018)
Colorectal cancer (CRC)	miR-324-5p (down)	TCGA database, 23 pairs of cancer tissues and ANCSs/mice	ITGA2	VPS9D1-AS1	VPS9D1-AS1 *via* miR-324-5p/ITGA2 could increase tumorigenesis.	Huang et al. (2022)
	miR-324-5p (down)	Mice	HMGXB3 and WASF-2	DICER	DICER/miR-324-5p *via* HMGXB3/WASF-2 targeting could impede CRC tumorigenesis.	Sun et al. (2017)
	miR-324-3p (down) in Ox-resistant tissues and cells	40 Ox-resistant and 40 Ox-sensitive CRC tissues/mice	ADAM17, Bcl-2, Cleaved caspase-3, E-cadherin and Vimentin	MALAT1	MALAT1 *via* miR-324-3p/ADAM17 axis regulation could enhance tumorigenesis, and Ox-resistance.	Fan et al. (2020)
	miR-324-3p (down)	—	ELAVL1, MMP-9, uPA, and uPAR	—	miR-324-3p *via* ELAVL1 suppression could prevent cell proliferation.	Xiao et al. (2019)
Endometrial carcinoma	miR-324-5p (down)	10 endometrial cancer and 10 normal endometrial specimens/mice	HMGA1	Circ0067835	Circ0067835 *via* miR-324-5p/HMGA1 axis could enhance cell proliferation.	Li u et al. (2020)
Gastric cancer (GC)	miR-324-5p (up)	30 pairs of GC tissues and ANCSs/mice	PTPRD	Circ0049447	Circ0049447 *via* miR-324-5p targeting could prevent tumorigenesis.	Tang et al. (2021a)
	miR-324-5p (down)	5 pairs of GC tissues and ANCSs/mice	HMGA1	Circ0091994	Circ0091994 through miR-324-5p/HMGA1 targeting could provoke tumorigenesis.	Xie et al. (2021)
	miR-324-3p (up)	68 pairs of GC tissues and ANCSs/mice	Smad4, β-catenin, cyclin D1, CD44, c-jun, c-Met, and TCF-1	—	miR-324-3p *via* Smad4/Wnt signaling pathway regulation could promote cell growth and migration, and repress apoptosis.	Sun et al. (2018)
	miR-324-5p (up)	122 pairs of GC tissues and ANCSs	PTEN	—	miR-324-5p *via* PTEN regulation could impede cell proliferation and provoke cell apoptosis.	Zheng et al. (2021)
Glioblastoma	miR-324-5p (down)	46 pairs of glioblastoma samples and normal brain specimens/mice	BCL2, PCNA, Ki67, cyclinD1, Bax, cleaved caspase3	SERPINE2	SERPINE2 *via* miR-324-5p/BCL2 regulation could promote cell proliferation.	Li et al. (2021)
Glioma	miR-324-5p (down)	TCGA and CGGA database, 43 pairs of glioma tissues and ANCSs/mice	KCTD20, CDK4, cyclin D1, Bcl2, Bax	NEAT1	NEAT1 through miR-324-5p/KCTD20 regulation could promote tumorigenesis.	Zhang et al. (2021)
	miR-324-5p (down)	CGGA database, 8 normal brain samples, 8 grade II and 8 grade IV glioma samples/mice	EZH2, DKK1 and p21	—	miR-324-5p *via* EZH2 targeting could hamper cell proliferation and TMZ resistance.	Zhi et al. (2017)
Hemangioma	miR-324-3p (down)	16 tissues from proliferative hemangiomas and 14 from involuting hemangiomas	PDRG1	FOXD2-AS1	FOXD2-AS1 *via* miR-324-3p/PDRG1 regulation could increase cell proliferation.	Zhao et al. (2020)
Hepatocellular carcinoma (HCC)	miR-324-5p (down)	Blood samples of 116 HCC patients, 43 liver cirrhosis patients, and 52 healthy controls	MMP2, MMP9, Bcl-2, cyclin D1, cyclin B1, c-myc	Lnc85	Lnc85 by miR-324-5p regulation could promote proliferation and migration.	Huang et al. (2020a)
	miR-324-5p (down)	46 pairs of HCC and normal tissues/mice	IGF2BP1	YY1, linc01134	YY1/linc01134/miR-324-5p/IGF2BP1 could participate in the HCC malignancy.	Rong et al. (2020)
	miR-324-5p (down)	11 pairs of HCC tumor and ANCSs	ETS1, SP1, MMP2, MMP9	—	miR-324-5p *via* ETS1 and SP1 targeting suppresses cell migration and invasion.	Cao et al. (2015)
	miR-324-3p (up in HBV-associated HCC)	Blood samples from 96 HBV-associated HCC cases, 52 HBV-unrelated HCC cases, 72 chronic hepatitis B patients, and 76 normal subjects	—	—	miR-324-3p participates in the progression of HBV-associated hepatitis to HCC.	Zhao et al. (2021b)
	miR-324-3p (up)	73 pairs of HCC tissues and ANCSs/mice	DACT1, β-catenin, c-Myc and cyclin D1	—	miR-324-3p *via* DACT1 targeting could promote cell viability.	Tuo et al. (2017)
	miR-324-5p (down)	42 airs of HCC tissues and ANCSs/mice	CDK16, JAK2/STAT3, Bax, Bcl-2, cleaved caspase3	GL63, CircZNF83	GL63 through regulation of and circZNF83/miR-324-5p/CDK16 axis and suppression of JAK2/STAT3 signaling pathway could prevent tumorigenesis.	Zhao et al. (2021a)
	miR-324-5p (down)	60 pairs of HCC and ANCSs/mice	ROCK1	LINC00491	LINC00491 *via* miR-324-5p/ROCK1 could induce proliferation, and inhibit apoptosis.	Wan g et al. (2021)
Lung cancer	miR-324-5p (up)	TCGA and GTEx project/100 pairs of lung cancer tissues and ANCSs	FBXO11	GATA6-AS1	GATA6-AS1 through regulation of miR-324-5p/FBXO11 and SNAIL could inhibit cell proliferation.	Wang et al. (2020b)
	miR-324-5p (-)	—	RAP1A, Bcl-2, cleaved caspase-3 and cleaved PARP	PCAT-1	PCAT-1 *via* miR-324-5p/RAP1A increases proliferation and inhibits apoptosis.	Huang et al. (2020b)
	miR-324 (up)	TCGA database	GPX3, RCAN1 and MGAT3	—	miR-324-5p promotes cell proliferation and invasion, while miR-324-3p only increases cell proliferation.	Lin et al. (2018)
Lung squamous cell carcinoma (LSCC)	miR-324-3p (up)	Plasma from 395 patients with different tumors and 195 healthy controls	331 genes	—	miR-324-3p level could act as an early detection and prognostic marker.	Gao et al. (2016)
	miR-324-3p (up)	TCGA/36 lung tissues	FAM46C	—	miR-296-5p, miR-324-3p, and miR-3928-3p could suppress FAM46C to induce MYC expression and cell proliferation.	Xia et al. (2018)
Non-small-cell lung cancer (NSCLC)	miR-324-3p (down)	40 pairs of lung adenocarcinoma specimens	Cyclin D1, ki-67	LOXL1-AS1	LOXL1-AS1 *via* downregulation of miR-324-3p enhances cell proliferation.	Xie et al. (2019)
Melanoma	miR-324-5p (down)	50 melanoma tissues and 50 normal skin tissues	CDK16	HCG18	HCG18 *via* miR-324-5p/CDK16 regulation could promote cell proliferation.	Zhang et al. (2022)
Multiple myeloma (MM)	miR-324-3p (-)	—	Wnt, β-Catenin, RUNX2 and ALP	LINC00461	LINC00461 reduces Wnt/β-catenin activity to inhibit the osteoblast differentiation through miR-324-3p targeting.	Wu et al. (2022)
	miR-324-5p (down)	Primary plasma cells of 26 MM patients and 2 healthy controls	BTRC, MTSS1, MMP2, MMP9, DZIP3, HECW2, UBR2, VHL, CBL	—	miR-324-5p could inhibit proliferative and migratory capacities of cells through suppression of ubiquitination pathway.	Zhang et al. (2018)
Nasopharyngeal carcinoma (NPC)	miR-324-3p (down)	54 NPC tissues and 34 normal and chronic inflammatory nasopharyngeal mucosal tissues	GLI3, E-cadherin, vimentin	—	miR-324-3p *via* GLI3 targeting could suppress cell proliferation.	Zhang et al. (2017)
	miR-324-3p (down)	39 Primary NPC and 21 normal nasopharyngeal epithelium (NPE) tissues	WNT2B, E-cadherin and Vimentin	—	miR-324-3p *via* WNT2B targeting could suppress migration and invasion of cells.	Liu et al. (2017)
	miR-324-3p (down)	GEO dataset, 76 pairs of NPC samples and ANCSs/mice	IL-6	SLC25A21-AS1	SLC25A21-AS1 *via* miR-324-3p/IL-6 axis could increase cell growth and MDR.	Wang et al. (2020a)
	miR-324-3p (-)	80 primary NPC tissues	SMAD7	—	miR-324-3p *via* SMAD7 targeting could reduce cell growth rate and promote apoptosis.	Xu et al. (2015)
Ovarian cancer	miR-324-5p (up)	—	GLI1	—	miR-324-5p downregulation could be considered as a modality for treatment of ovarian cancer	Suardi et al. (2020)
	miR-324-3p (down)	TCGA database/mice	LY6K, E-cadherin, N-cadherin and vimentin	ZNF252P-AS1	ZNF252P-AS1 *via* miR-324-3p/LY6K targeting could promote cell proliferation, invasion, migration, and EMT, but prevent apoptosis.	Geng et al. (2022)
Pancreatic cancer (PC)	miR-324-3p (down)	GEO database/40 pairs of PC tissues and ANCSs	Bcl-2, Bax and Cleaved caspase-3	LINC01320	LINC01320 *via* miR-324-3p targeting could inhibit growth, migration and invasion.	Meng et al. (2021)
	miR-324-5p (up)	18 pairs of PC tissues and ANCSs/mice	KLF3, PCNA, BAX	—	miR-324-5p *via* KLF3 silencing could promote tumorigenesis.	Wan et al. (2020)
Papillary thyroid cancer (PTC)	miR-324-5p (up in LNM+)	41 PTC tissues (LNM+, −) and 143 FNA samples (LNM+, −)	—	—	miR-324-5p increases proliferation, migration and invasion of cells but inhibits apoptosis.	Yang et al. (2017)
	miR-324-3p (down)	GEO and TCGA database/40 pairs of PTC tissues and ANCSs	LASP1	MIAT	MIAT by miR-324-3p/LASP1 targeting could provoke PTC proliferation and invasion.	Liu et al. (2019a)
	miR-324-5p (up)	—	PTPRD	—	miR-324-5p *via* PTPRD/CEBPD axis could participate in the progression of cancer *via* VEGF.	Yang et al. (2020)
Prostate cancer (PCa)	miR-324-5p (down)	TCGA database/57 PCa tissues/mice	TGFBR1	PCAT7	PCAT7 *via* regulation of miR-324-5p/TGFBR1 axis and activation of TGF-b/SMAD signaling could increase cell migration, invasion, and EMT and bone metastases.	Lang et al. (2020)

### The impact of miR-324 on therapeutic response

In addition, miR-324 participates in the response of cancer cells to therapeutic agents. For instance, according to Wu et al. study on HEK-293, PC9, HCC827 cell lines and mice model of lung cancer, lncRNA APCDD1L-AS1 *via* regulation of miR-1322/miR-1972/miR-324-3p/SIRT5 axis could cause upregulation of EGFR and induce resistance to icotinib (Wu et al., 2021).

### The prognostic role of miR-324 in cancers

The impact of dysregulation of miR-324 in tumor tissues on patients' survival has been assessed through Kaplan-Meier analysis. Moreover, multivariate Cox analysis has shown association between expression levels of miR-324 and clinical data (Table 2). For instance, miR-324 has been among miRNA whose expression levels in breast cancer tissues have been associated with tumor size or lymph node involvement depending on the status and expression levels of hormone receptors, HER2, and Ki-67 (Kalinina et al., 2021). In bladder cancer, over-expression of miR-324-5p has been associated with clinical stage. Besides, significant associations have been observed between high miR-324-3p and miR-324-5p expression levels and poor overall survival (Tsai et al., 2020). On the other hand, in glioma samples, lower level of miR-324-5p has been associated with high grade (grade III and grade IV) (Zhi et al., 2017).

**TABLE 2:** Prognostic role of miR-324 in cancers (OS, overall survival; DFS, disease free survival).

Samples	Kaplan meier	Multivariate Cox analysis	References
414 bladder cancer tissues	High miR-324-5p and 5p expression levels were correlated with OS.	Over-expression of miR-324-5p was associated with clinical stage. A significant association was detected between miR-324-3p and 5p expressions and poor OS.	Tsai et al. (2020)
45 breast cancer tissues	—	Expression of miR-324-3p was associated with tumor size and TNM stage.	Zhang et al. (2019a)
156 breast cancer tissues	—	miR-324 expression was associated with tumor size in luminal B HER2-negative tumors. Upregulation of miR-324 was associated with age <50 and HER2 + status in ER+ and/or PR+ tumors with low Ki-67.	Kalinina et al. (2021)
TCGA database for esophageal squamous cell carcinoma, and esophageal adenocarcinoma	High expression of miR-324-5p was related to poor prognosis and OS.	—	Chen et al. (2021a)
68 gastric cancer tissues	—	Expression level of miR-324-3p was associated with tumor size.	Sun et al. (2018)
122 gastric cancer tissues	Overexpression of miR-324-5p was associated with poor OS.	Over-expression of miR-324-5p was associated with lymph node metastases and advanced TNM stage.	Zheng et al. (2021)
CGGA database, 8 grade II and 8 grade IV glioma tissues	Low level of miR-324-5p was associated with poor survival	Lower level of miR-324-5p was associated with high grade.	Zhi et al. (2017)
Blood samples from 96 HBV-related HCC patients	High serum level of miR-324-2p in HBV-related HCC patients was associated with poor OS.	Serum levels of miR-324-2p in HBV-related HCC patients were associated with cirrhosis, tumor size, and clinical stage.	Zhao et al. (2021b)
73 HCC tissues	Upregulation of miR-324-3p was associated with shorter OS and DFS.	Upregulation of miR-324-3p was associated with larger tumor size, multiple tumoral nodules and higher TNM stage.	Tuo et al. (2017)
Primary plasma cells of 26 MM patients	—	Expression of miR-324-5p was negatively associated with stage.	Xia et al. (2018)
54 Nasopharyngeal carcinoma (NPC) tissues	Higher level of miR-324-3p was associated with longer survival time.	Expression level of miR-324-3p was associated with level of differentiation, TNM stage, and lymph node metastases.	Zhang et al. (2018)
39 NPC tissues	—	Lower level of miR-324-3p was associated with tumor T classification, clinical stage and lymph node metastases.	Zhang et al. (2017)
80 NPC tissues	Low expression of miR-324-3p was associated with poor OS and recurrence-free survival	Low expression of miR-324-3p was associated with EBV infection, advanced clinical stage, and high rates of radioresistance.	Liu et al. (2017)
OV TCGA database	Over-expression of miR-324-3p was related with higher survival rate.	—	Xu et al. (2015)
TCGA database for LSCC	Over-expression of miR-324-3p was related to with worse prognosis.	—	Geng et al. (2022)

## Role of miR-324 in non-malignant disorders

miR-324 has been among dysregulated miRNAs in aneurysmal subarachnoid hemorrhage (SAH) patients. Notably, miR-324-3p has been shown to be upregulated in SAH patients with delayed cerebral infarction (DCI) as well as non-DCI group. Yet, no significant difference has been detected in expression levels between patients with and without DCI (Su et al., 2015). Another investigation has revealed importance of miR-324-3p in the pathogenesis of anorectal malformations and demonstrated that Rno_circ_0005139 can increase cell proliferation and apoptosis *via* influencing activity of miR-324-3p/Wnt5a axis (Liu et al., 2020a). In the context of cardiac disorders, NFAT4 has been shown to regulate miR-324-5p/Mtfr1 axis to enhance mitochondrial fission and cardiomyocyte apoptosis and aggravate pathogenic events in the myocardial infarction (Wang et al., 2015). Table 3 summarizes the impact of miR-324 in the pathogenesis of non-neoplastic conditions.

**TABLE 3:** miR-324 in non-malignant diseases (DCI, Delayed cerebral infarction; IEC, intestinal epithelial cells; MCD, minimal change disease; HG, high glucose; Ucn, Urocortin; OGD/R, oxygen-glucose deprivation/reoxygenation; NOF, neck of the femur; EX, aerobic exercise; SED, sedentary).

Disease	Expression	Samples	Downstream targets	Other related molecules	Description	References
Aneurysmal subarachnoid hemorrhage (SAH)	miR-324-3p (up)	Blood samples from 20 SAH with DCI, 20 without DCI, and 20 controls		—	miR-324 could be considered as a potential biomarker of SAH.	Su et al. (2015)
Anorectal malformation	miR-324-3p (up)	Rat	Wnt5a	Rno-circ_0005139	Rno_circ_0005139 *via* miR-324-3p/Wnt5a targeting could increase cell proliferation.	Liu et al. (2020a)
Cardiac disease	miR-324-5p (down)	Mice	Mtfr1, caspase-3	NFAT4	NFAT4 through miR-324-5p/Mtfr1 axis could increase mitochondrial fission, cardiomyocyte apoptosis and myocardial infarction.	Wang et al. (2015)
Diabetic nephropathy (DN)	miR-324-3p (down in DN)	Renal tissues from 10 MCD patients and 9 DN/rat	DUSP1, collagen I, collagen IV, fibronectin	NR-038323	NR-038323 induced by HG *via* miR-324-3p/DUSP1/p38MAPK and ERK1, 2 regulations could mitigate renal fibrosis.	Ge et al. (2019)
Epilepsy	miR-324-5p (-)	Mice	Kv4.2	—	miR-324-5p *via* Kv4.2 targeting could decrease A-type potassium currents and increase the frequency of seizures.	Tiwari et al. (2019)
Epilepsy	miR-324 (-)	Mice	Suox and Cd300lf	—	miR-324 *via* Suox and Cd300lf targeting could play role in neurological disease	Hayman et al. (2021)
HIV lipodystrophy	miR-324-5p (down in HIV lipodystrophic cases)	subcutaneous abdominal fat from 11 persons with HIV, 9 with and 9 without lipodystrophy, and 9 uninfected individuals/mice	Ltbp2, Wisp2, and Nebl	—	miR-324-5p absence and Ltbp2 suppression could cause dysregulation in different adipose differentiation and inflammation markers which led to HIV lipodystrophy.	Srinivasa et al. (2021)
Idiopathic pulmonary fibrosis (IPF)	miR-324-5p	Blood samples from 10 IPF patients and 10 healthy controls	13 different genes	Circ100906	Circ100906 *via* miR-324-5p targeting could participate in IPF pathogenesis.	Li et al. (2018)
Influenza A	miR-324-5p (down)	GEO database/nasopharyngeal swabs from 4 healthy individuals and 9 patients with swine influenza (H1N1)	PB1, CUEDC2, type I interferon, type III interferon, and ISGs	—	miR-324-5p could suppress H5N1 replication by targeting of PB1and cellular CUEDC2 gene.	Kumar et al. (2018)
Ischemic stroke (IS)	miR-324-5p (down)	GEO database, Blood samples from 80 cases of acute ischemic stroke patients and 80 cases of healthy controls/rat	RAN, caspase-3	—	In ischemic stroke, miR-324-5p downregulation and subsequently RAN upregulation could prevent neuronal cell proliferation and glucose uptake and promote apoptosis.	Gu et al. (2020)
IS	miR-324-3p (down)	Rat	VEGFA	SNHG11	Dexmedetomidine *via* regulation of SNHG11/miR-324-3p/VEGFA pathway and oxidative stress markers could improve neurological trauma after OGD/R.	Chen et al. (2021b)
Tuberculosis (TB)	miR-324-5p (down)	50 pulmonary TB patients and 50 healthy cases	CTLA4	Circ0003528	Circ0003528 through miR-324-5p/CTLA4 axis could cause tuberculosis related macrophage polarization.	Huang et al. (2020c)
Myocardial ischemia reperfusion (I/R) injuries	miR-324-3p (down)	Rat	BRCA1, BIM, STAT2, PDE4a, CASQ1, NFAT5, XBP1, MAP3K12, CPT2, FoxO1, MTRF1, TAZ	—	Ucn-1, 2 prescriptions *via* miR-324-3p upregulation and downregulation of various genes associated with apoptosis could exert protective effects on heart.	Díaz et al. (2017)
Neurological damages	miR-324-5p (down)	Mice	CCL5,p-CREB, p-ERK1/2	—	miR-324-5p downregulation or CCL5 upregulation associated with dicer deficiency *via* MAPK/CREB signaling pathway suppression could exacerbate neurodegeneration.	Sun et al. (2019)
Nerve injury	miR-324-3p (-)	Rat	VEGFB	CircAnks1a	CircAnks1a *via* miR-324-3p/VEGFB axis could augment hypersensitivity to pain.	Zhang et al. (2019b)
Osteoarthritis (OA)	miR-324-5p (up)	OA cartilage from patients with joint replacement, healthy cartilage from patients with fracture of the NOF/mice	GLI1, SMO in human, Gpc1in mice	—	The regulatory function of miR-324-5p on hedgehog pathway is species- dependent.	Woods et al. (2019)
Parkinson disease (PD)	miR-324 (down in EX-PD group)	Rat	Vdac1	—	In EX-PD group, downregulation of miR-324 *via* CaMK/mTOR signaling pathway regulation could influence UCH-L1 level and repress progression of PD.	Liu et al. (2019b)
Polycystic ovarian syndrome (PCOS)	miR-324 (down)	15 PCOS and 15 normal ovarian tissues	WNT2B, Bax, caspase-3, and Bcl-2	—	miR-324 over-expression *via* WNT2B targeting could promote cell apoptosis.	Yuanyuan et al. (2019)
Myocardial infarction (MI)	miR-324-5p (-)	Rat	TOLLIP	—	Upregulated miR-324-5p *via* TOLLIP targeting could increase proliferation, migration and post-MI myocardial repair.	Ji et al. (2021)
Pulmonary arterial hypertension (PAH)	miR-324-5p (down)	Blood samples from 14 healthy volunteers and 12 IPAH patients/lung tissue from 6 PAH patients and control tissues from 6 patients with bronchial carcinoma/mice	Notch4, ETS-1, caspase3/7	—	KLF2-induced miR-324-5p reduces proliferative and angiogenic responses and disease progression.	Sindi et al. (2020)
Pulmonary inflammatory disease	miR-324-3p (up)	—	IKβ-α, IKKβ, IL-6, IL-8, TNF-α, p-P65, ICAM-1, and VCAM-1.	H19	Dexamethasone *via* H19/miR-324-3p axis could decrease pulmonary inflammation.	Chen et al. (2021c)
Renal fibrosis	miR-324-3p (up)	Rat	Prep	ACE	ACE inhibition *via* miR-324-3p/Prep/Ac-SDKP axis could alleviate fibrosis.	Macconi et al. (2012)
Renal fibrosis	miR-324-3p (up)	6 normal, 11 mild fibrosis, and 13 moderate fibrosis tissues/mice	NRG1, ATG5, ATG7, LC3II, and LC3I, P62, Collagen I, Fibronectin, and a-SMA	LncRNA 74.1	LncRNA 74.1 *via* regulation of miR-324-3p/NRG1/PI3K/AKT signaling pathway could promote autophagy and mitigate fibrosis.	Tang et al. (2021b)
Seizure	miR-324-5p (up)	Mice	Kv4.2	—	miR-324-5p downregulation and Kv4.2 enhancement could diminish seizures.	Gross et al. (2016)
Thermal injury and burns	miR-324-5p (up)	—	CDK16	TPT1-AS1	TPT1-AS1 through miR-324-5p/CDK16 axis could increase cell viability and ECM production while hamper apoptosis.	Luo et al. (2021)

Liu et al. (2021) have examined miR-324-3p expression levels in primary Granulosa cells (GCs) retrieved from the follicles of ovarian tissue in the follicular phase from high- and low-yielding goats and found that upregulation of chi-miR-324-3p can inhibit GCs proliferation *via* targeting DENND1A gene and consequently downregulating expression of GCs proliferation markers such as LHR, cyclin D2, and CDK4 (Liu et al., 2021).

The importance of miR-324 in skeletal muscle differentiation has been investigated in different studies. According to Liu et al. (2020b) study in mice models, mouse C2C12 myoblasts and human HEK293T cell line, miR-324-5p has been over-expressed in skeletal muscle. Through targeting lncDum and Pm20d1, this miRNA could inhibit myoblasts differentiation and lipid aggregation (Liu et al., 2020b).

In the bone remodeling process in the body, small extracellular vesicles (sEVs) secreted by mature osteoclasts play an important role. One of the upregulated miRNAs in sEVs is miR-324. This miRNA that could cause differentiation and mineralization of bone progenitor cells by through silencing of ARHGAP1 and subsequent stimulation of RhoA/ROCK pathway (Liang et al., 2021).

According to miRNome analysis in muscle specimens of spinal muscular atrophy patients and controls and *in vitro* and *in vivo* experiments, miR-324-5p has been identified as a differentially expressed miRNA. So, in addition to the assessment of SMN2 copy number, measurement of this miRNA in SMA patients can be helpful in prognosis anticipation (Abiusi et al., 2021).

## Discussion

Dysregulation of miR-324 has been described in a variety of tumor types and cancer cell lines. Most of studies have stated downregulation of miR-324 in these samples. However, some inconsistencies exist in certain types of cancers. For instance, both miR-324-3p and miR-324-5p have been reported to be upregulated in bladder cancer. In liver and cervical cancers, miR-324-5p is downregulated, while miR-324-3p is upregulated. In contrast, in ovarian, thyroid and pancreatic cancers, miR-324-5p has been found to be upregulated, while miR-324-3p has been down-regulated. Finally, in gastric and lung cancer, no consistent pattern has been reported. Thus, although the observed inconsistencies in expression pattern and function of miR-324 might be explained by different functions of miR-324-5p and miR-324-3p, tissue-dependent factors are also involved.

Several lncRNAs and circular RNAs such as LINC00963, LINC01320, LINC00461, LINC00491, LINC01134, LINC00511, SNHG22, H1FX-AS1, TPT1-AS1, VPS9D1-AS1, MALAT1, NEAT1, FOXD2-AS1, Lnc85, GATA6-AS1, PCAT-1, LOXL1-AS1, SLC25A21-AS1, ZNF252P-AS1, MIAT, PCAT7, Circ0067835, Circ0049447, Circ0091994 and CircZNF83 have been shown to act as a sponge for miR-324 and decrease its bioavailability. Cumulatively, changes in the expression of these molecules are the most appreciated route of regulation of expression of miR-324. Future studies are needed to find other mechanisms of dysregulation of miR-324 in different tissues.

Additionally, miR-324 has important functions in the pathogenesis of a number of non-malignant conditions such as pulmonary and renal fibrosis as well as I/R injuries. Based on the high burden of ischemic stroke and myocardial infarction on the public health, further assessment of the role of miR-324 in these conditions can facilitate design of appropriate preventive or curative strategies. In SAH, significant upregulation of miR-324 has indicated that this miRNA can be a potential biomarker. Most notably, this miRNA has high accuracy for differentiation of SAH patients from healthy controls (AUC values of 0.97 and 0.96 for DCI, non-DCI groups, respectively) (Su et al., 2015). Moreover, miR-324 has been found to be up-regulated in osteoarthritis cartilage and regulate Hh signaling. Since, miR-324-5p has been shown to regulate osteogenesis in human mesenchymal stem cells (Woods et al., 2019), it can be regarded as a possible target for therapeutic modalities for osteoarthritis. Additionally, miR-324-5p expression has been shown to be altered in the brain samples of suicide victims with depression (Smalheiser et al., 2012) and in the amygdala in posttraumatic stress disorder (Balakathiresan et al., 2014), indicating the importance of this miRNA in the pathogenesis of neuropsychiatric conditions and its possible role as a biomarkers for this kind of disorders.

Cumulatively, miR-324 is a candidate for design of novel therapeutic strategies for neoplastic and non-neoplastic conditions, since it has been found to be dysregulated in a variety of disorder. Moreover, *in vitro* and *in vivo* studies have shown that amelioration of miR-324 levels can reverse pathologic events occurred in these disorders. However, tissue-specific expression and function of this miRNA should be considered before introduction of any treatment modality in clinical settings.
